# Contribution of education, occupation and cognitively stimulating activities to the formation of cognitive reserve

**DOI:** 10.1590/S1980-57642009DN20300003

**Published:** 2008

**Authors:** Beatriz Baldivia, Vivian Maria Andrade, Orlando Francisco Amodeo Bueno

**Affiliations:** 1 Psychologist, MD, Department of Psychobiology, Universidade Federal de São Paulo, São Paulo SP, Brazil.; 2 Psychologist, PHD, Department of Physiology, Universidade Federal de Sergipe, Sergipe, Brazil.; 3 Adjunct Professor, PHD, Department of Psychobiology, Universidade Federal de São Paulo, São Paulo SP, Brazil.

**Keywords:** cognitive reserve, aging, Alzheimer’s disease, occupation attainment, schooling, cognitive stimulation

## Abstract

The cognitive reserve (CR) concept posits that there is individual variability in processing task demands and coping with neurodegenerative diseases. This variability can be attributed to the protective effects derived from continuous cognitive stimulation throughout life, including formal education, engagement in cognitively stimulating activities and occupation. These can result in protection against age-related cognitive decline and reduce the risk of developing Alzheimer’s disease. The aim of this review is to summarize the main features of CR formation and to discuss the challenges in carrying out CR research in developing countries.

## Abstract (pt)

O conceito de Reserva Cognitiva (RC) postula que há variabilidades individuais no processamento de tarefas e no enfrentamento de doenças neurodegenerativas. Essa variabilidade pode se causada pela conseqüência do envolvimento em estimulações cognitivas sistemáticas ao longo da vida, tais como escolaridade, ocupação profissional e engajamento em atividades cognitivamente estimulantes; resultando na proteção contra os declínios cognitivos relacionados à idade e na diminuição do risco de desenvolver Doença de Alzheimer. O objetivo desta revisão é apresentar os principais fatores formadores de RC e discutir os desafios de realizar pesquisas sobre RC em países em desenvolvimento.

The idea of a reserve against brain damage arose from the repeated observation that there is no direct relationship between the degree of brain pathology or brain damage and the clinical manifestation of such damage.^1^ In 1988, Katzman et al.^2^ described ten cases of elders who were only found to have advanced Alzheimer’s disease (AD) on *post mortem* exam. This lack of a relationship between severity of clinical symptoms and degree of brain damage was attributed to the above-average brain size of these patients. Therefore, something must be mediating the relationship between the extent of pathology and clinical outcome, and the reserve concept has been proposed to explain this phenomenon.

The reserve concept is applicable to any situation in which the brain sustains pathology, and is related to the degree of pathological processes that the brain is able to handle before overt clinical manifestation. In addition, the reserve concept suggests that there is individual variability in the way people process cognitive information, perform mental tasks and cope with neurodegenerative diseases.^3^

Two related models have been proposed to explain the reserve concept: the active model and the passive model.

## 1. Passive models

The passive model – or cerebral reserve – is defined as the amount of brain damage that can be sustained before clinical expression of a disease. The threshold model was revised by Satz in 1993^4^ and is one of the most accepted passive models. It implies the concept of brain reserve capacity (BRC), related to measures including brain size or number of neurons. This model recognizes that there are individual differences in BRC and that specific deficits emerge once BRC is depleted beyond its threshold.^1,3^ For example, if two patients have the same lesion extent but different levels of BRC, the clinical deficit will only appear in the patient with the lower BRC level given that the lesion exceeds the threshold of the BRC. An individual with greater BRC however, might be unaffected if the threshold is not exceeded. Thus, BRC level has a linear relationship with a protective factor against cognitive impairment, at least up to the point that brain damage crosses a certain threshold.

Therefore, the threshold and BRC models are considered to be passive models of reserve because 1) they postulate that there is a common functional impairment cut-off for everyone, and 2) they are essentially quantitative models. Individual differences are related to BRC level, and clinical expression depends on whether the BRC level is depleted or not. The model does not account for individual differences in how the brain processes cognitive tasks or how it copes with lesions.^3^

Cerebral reserve may be a passive capacity of the brain, serving to withstand brain aging or injury, beyond which clinical deficits appear. For example, if brain size or number of neurons is a proxy for passive cerebral reserve, the cerebral reserve hypothesis posits that those with larger brains or a greater number of neurons are able to withstand greater insults to the brain than those with smaller brain volumes. It is difficult to test the cerebral reserve hypothesis because it would require the use of measures that are rarely available, including baseline data with which to compare current cognitive status. Staff et al.^5^ tested the influence of education, head size and occupation as proxies of reserve in volunteers whose cognitive function was tested at ages 11 and 79 years. The authors found that education and occupation, but not total intracranial volume, contributed to cerebral reserve, minimizing the effects of age-related cognitive changes. On the other hand, Mortimer et al. found interaction of smaller head circumference with lower education to be associated with the presence of dementia in Catholic sisters, controlling for age and the presence of one or more apolipoprotein E-e4 alleles.^6^

## 2. Active models

The active models of reserve suggest that the brain actively attempts to compensate for environmental tasks. Stern et al.^7^ proposed that in this kind of model, at least two kinds of reserve can be demonstrated: cognitive reserve and neuronal compensation. The recruitment of complementary areas of the brain is a natural response to increasing task demands, making it possible for healthy people (cognitive reserve) as well as those with brain damage to utilize this strategy (neuronal compensation).

The neurophysiological substrates of cognitive reserve (CR) postulate that there is a natural variability among healthy subjects in their ability to recruit different pathways to cope with task demands. Compensation involves the recruitment of brain structures or networks not normally used by healthy individuals in order to compensate for and minimize the impact of brain damage.

### 2.1. Cognitive reserve (CR)

Cognitive reserve can also be defined as the ability to optimize or maximize performance through differential recruitment of brain networks. This can reflect the use of alternate cognitive strategies.^3^ That is, if a person has a high level of cognitive reserve, they could sustain higher levels of pathology by using alternate brain networks or cognitive strategies. The definition of CR allows these two possibilities: differences in recruitment of the same network and the use of alternate pathways.

### 2.2. Neural compensation

Neural compensation represents a change that is induced by brain damage, which cannot be a natural answer to the degree of difficulty of a task. Neural compensation implies an effort to maximize performance in the event of brain damage by using alternate areas and network pathways.^7^

## 3. Neuroimaging evidence for CR and neural compensation

Data from imaging studies have served as an indirect measure of CR and have provided the foundation of the first bids to correlate neural function and CR. Considering that the level of CR reflects differences in how tasks are processed, functional imaging should be able to capture these differences.^7^ Some studies have investigated CR-mediated differential brain activation using diverse conditions. Differential activation patterns were found to be correlated to CR level in younger volunteers, showing that those with a lower level of CR activated more brain regions than those with a higher level of CR in a visual recognition task.^8^ Comparisons between young and older healthy people also showed different patterns of activation according to age^9-11^ as a function of CR.^8^ A number of studies have shown that healthy elders with additional activation of areas contralateral to those activated by younger subjects, had better performance than elders who did not activate additional areas, suggesting a compensatory strategy^12^ to cope with age-related changes. This was also observed in AD patients.^8,9,11^ Cerebral blood flow is a good surrogate for AD pathology, with lower flow or metabolism indica tive of more advanced pathology.^13^ In AD patients, those with higher pre-morbid intellectual ability,^13^ occupation,^14^ education^14^ and engagement in cognitively stimulating activities^16^ had more metabolic deficits when controlling for clinical severity. This suggests that higher CR can result in milder clinical deficits, despite comparable pathology, supporting the notion that individuals with greater CR can tolerate greater pathology.

The relationship between brain pathology in AD (amyloid plaques and neurofibrillary tangles) and educational level suggests that education can modify the relationship of amyloid plaques, but not tangle load. Recently, Kemppainen et al.^16^ used brain amyloid ligand^11^ C-labeled Pittsburgh Compound B ([^11^C] PIB) uptake to indicate amyloid accumulation and compare differences in cognitive performance of high and low-educated patients with mild AD. They found that high-educated patients showed increased ([^11^C] PIB) uptake in lateral frontal cortex and lower glucose metabolic rate in the temporoparietal cortical regions compared with low-educated patients. These results suggest more advanced pathological and functional brain changes in high-educated patients with mild AD, and corroborate the cognitive reserve hypothesis, i.e., delayed clinical expression of the disease in this group. Moreover, this new finding suggests that brain cognitive reserve can partly compensate for the effects of amyloid plaque accumulation in cognition and point to the importance of developing reliable markers of underlying AD pathology for early AD diagnosis.

Structural imaging studies are also a useful method of investigating the CR hypothesis regarding environmental demands. For example, Maguire et al. found that taxi drivers show topographic hippocampal reorganization, correlated with their length of time on the job, which favors visuospatial learning^17^ and is in turn correlated to increased gray matter volume.^17^

Solé-Padullés et al.^19^ measured CR (a combination of an occupation-education scale, engagement in intellectual and social activities and premorbid IQ) in healthy elders and in those with mild cognitive impairment (MCI) and mild AD, who underwent MRI and functional MRI exams. The authors found that among healthy elders, higher CR was associated with a larger brain and reduced activity during cognitive processing, suggesting more effective use of cerebral networks. The results from healthy elders showed an inverse effect of CR measures on both brain function and structure: higher CR was associated with reduced brain activity during cognitive performance, while the opposite was observed among AD and MCI groups.

## 4. Aging and cognitive reserve

Aging entails a pattern of mild cognitive impairments^20^ which are strongly predicted by cognitive ability during childhood, accounting for about 50% of inter-individual variance in later life.^21^

Healthy aging is accomplished by the maintenance or improvement of abilities such as knowledge about the world, vocabulary and processes based on crystallized abilities,^22^ while general abilities decline.^23^

Two approaches have emerged to explain these age-related cognitive changes: the common cause hypothesis and the specific gain/loss hypothesis.^24^ According to the first approach, performance on a variety of tasks depends on component processing speed, which declines with age^25^ leading to global changes in cognitive performance. In contrast, the specific gain/loss or frontal-executive hypothesis suggests that frontal lobe functional changes can be explained as a result of neurobiological changes in this region,^26,27^ which influence executive control on other cognitive functions (e.g. aspects of memory function).^28^ Structural and neuronal network changes are also observed during healthy aging.^29,30^ Neuronal loss within prefrontal cortex areas ^31^ and atrophy within frontal cortex^32^, and to a lesser degree within parietal^33^ and temporal cortices^34^, are commonly observed. Atrophy in the hippocampus and amygdala is related to the risk of developing AD,^35^ while decreasing thalamic volume is related to lower performance on speed processing tasks.^36^

Despite this, phenomena such as neuroprotection^37^ and neurorestoration^38^ allow the brain to repair itself, adapt or even compensate for neuronal loss and age-related cognitive decline. Exposure to – and interaction with – a rich environment influences these processes of neuroplasticity^39^ and is related to the rate of neurogenesis in both young and older animals.^40^ In humans, those with higher levels of intellectual ability, education and socioeconomic status are more likely to develop an engaging lifestyle, which in turn contributes to the maintenance of their previous level of functioning during healthy aging. There is also evidence to suggest that environmental enrichment might act directly in preventing or slowing the accumulation of AD pathology.^42^

### 4.1. Cognitive reserve and Alzheimer disease

Alzheimer disease (AD) is a primary cause of dementia, corresponding to about 60% of cases.^43^ AD has been broadly studied for a better understanding of the underlying mechanisms of CR, given that it progressively affects cortical areas that carry out diverse cognitive functions, allowing for insight into the protective mechanisms of CR.

Incidence studies are an appropriate method for assessing the burden of dementia and investigating risk factors and other variables that could influence incidence rate.^44^ Unlike developed countries, few studies have been conducted on the incidence^45^ of dementia in developing countries. The heterogeneity of educational and socioeconomic levels in the population of developing countries could provide more information about the relative importance of ethnicity and social factors in the development of dementia.^44,46^ The prevalence of dementia in developing countries is also scarce,^47^ but the foundation of the 10/66 Dementia Research Group^48^ was founded to bridge this gap and minimize methodological difficulties.

The relationship between low educational level and AD incidence has been reported in some Brazilian studies.^49-51^ Nitrini et al.^44^ found a statistical trend toward a higher incidence of dementia in illiterate individuals, but multivariate analysis did not confirm this result. These controversial findings point to low education level as a risk factor for dementia, which is also related to other risk factors such as nutrition, access to health services and socioeconomic level^51^. Nitrini et al.^44^ found no effects of socioeconomic level on the incidence of dementia; however, this could be explained by not taking into account socioeconomic level during childhood or early adulthood, when factors such as diet and quality of health care could have a greater impact on the risk of developing dementia in the future.

One possibility is that the higher prevalence of dementia seen in individuals with a lower education level could be the result of detection bias, due to the low sensitivity of neuropsychological tests when administered to this population^52^. Changes in performance that disrupt daily activities however, have also been reported in those with fewer years of schooling.^53^

Although high educational level is associated with a lower incidence of AD and might lead to greater reserve, the outcomes of AD for these patients are worse. In a prospective study of AD patients matched for clinical severity at baseline,^14^ those with higher educational or occupational level died sooner than those with lower levels. Although Geerlings et al.^54^ did not observe this finding, a follow-up study found the same result.^55^ In a longitudinal study of memory decline in AD, more rapid decline was detected in Alzheimer patients with a higher educational level.^56^

The CR hypothesis posits that at any given level of clinical severity, the underlying pathology of AD is more advanced in patients with a higher CR level; the protective effect of CR allows these patients to better cope with the pathology before they begin to display clinical symptoms.^2,15^ When the pathology becomes very severe however, there is no longer a substrate for cognitive reserve to act within, and cognitive decline accelerates.^6,56-58^

Education is a factor that may delay the manifestation of symptoms in individuals with AD pathology, possibly by allowing them to use cognitive processing or compensatory approaches which enable them to cope better with brain damage.^6^ Highly educated individuals thus have a shorter duration of diagnosed disease before death.^14^

## 5. Variables in the formation of cognitive reserve

The development of CR is associated with exposure to, and interaction with, favorable environments, and is also associated with genetic predisposition (for a review of the genetic aspects, see Lee^59^). The next section will discuss the contribution of education, engagement in cognitively stimulating activities, occupation, and related variables to the formation of CR.

### 5.1. Education

Several studies have reported an association between education and the prevalence of dementia, including some Brazilian studies.^49,50^ Herrera et al.^50^ found a prevalence of dementia which ranged from 3.5% among elders with eight or more years of schooling to 12% among illiterate individuals. They also found that education level was independently associated with higher prevalence of dementia.^50^

Nitrini et al.^44^ found no effects of present socioeconomic level, and observed a trend toward a higher incidence of dementia in illiterates, but this was not confirmed by multivariate analysis.

Bottino^51^ studied the prevalence of dementia and associated factors in a community sample from the city of São Paulo and found that age over 69 years, illiteracy, minimal education (4 years or less), stroke, head trauma and diabetes were all identified as potential risk factors, while reading books was a potential protective factor.

There is also evidence for the role of education in age-related cognitive decline. Several studies on healthy aging have reported that individuals with a low educational level have an accentuated decline in memory,60-61 verbal skills62-63 and functional level.64 Le Carret et al.^65^ also found that a higher level of education increased processing and conceptualization ability and could delay the clinical expression of neurodegenerative illnesses by maintaining global cognitive efficiency.

This evidence suggests that the same education-related factors that delay the onset of dementia might also allow individuals to cope more effectively with the brain changes that accompany healthy aging.

The effects of education on neuropsychological test performance are not linear. Differences are more prominent when a group of illiterate subjects is compared to a group with three years of schooling, while differences are smaller when groups with higher levels of education are compared. Therefore, the effects of education represent a kind of negatively accelerated curve which tends to plateau. This occurs because the ceiling in neuropsychological tests is low, reinforcing the idea that education might represent the most significant variable in neuropsychological test performance.^62^

Education level has been widely used as a proxy for reserve, probably because it is relatively easy to ascertain and is the product of ability or experience.^6^ In passive models of reserve,^3^ education is a proxy for the brain’s capacity (synaptic density or complexity) to tolerate a gradual insult. In active models, years of education would be an indicator of the brain’s ability to compensate for pathology with more efficient use of existing cognitive networks, or recruitment of alternate networks.

Alexander and colleagues (1997)^67^ suggested that an estimate of IQ or a measure of pre-morbid IQ might be a powerful measure of reserve. On the other hand, education – in addition to other life experience – probably impacts reserve more than maximizing innate intelligence. There are a number of ways, however, in which cultural, racial and economic factors can affect the predictive power of this proxy.^68^ For example, years of education may not be an accurate representation of native ability among minority or immigrant elders because they may not have achieved a high educational level due to limited opportunities or negative socioeconomic or environmental influences, such as racism, segregation and poverty.^68^ Moreover, taking years of schooling as a measure of CR implies that the quality of the educational experience is the same for all individuals, which is clearly not a valid assumption.

Thus, literacy measures of educational experience could be more accurate than years of education.^69^ Literacy involves not only the ability to read and write script, but also the knowledge of how and in what context to apply literacy skills for specific aims. It therefore constitutes a superior assessment of the knowledge, strategy and skills needed to perform well on traditional neuropsychological tasks.^69^

The role of education in cognition is not fully elucidated, but its contribution could be related to amount of time spent engaging in cognitively stimulating activities^70,71^. Katzman^72^ suggested that education could increase synaptic density and promote an intellectual and creative activity pattern, resulting in lifelong neuronal activity that could be physiologically beneficial.

### 5.2. Participation in cognitively stimulating activities

Cognitive activities are those in which seeking or processing information is central to participation in the activity.^73^ Frequent participation in cognitively stimulating activities has been hypothesized to maintain a higher level of cognitive function during healthy aging^74^ and to reduce the risk of AD.^75^

Wilson et al.^76^ tested the association between engagement in cognitively stimulating activities and the incidence of AD and decline in cognitive function, in a large cohort of older catholic clergy members who were examined annually for up to 7 years. The authors found that a 1-point increase in cognitive activity (measured by the number of activities involving information processing as a central component and by the frequency of participation in each activity) was associated with a 33% reduction in risk of AD. They found no evidence that frequency of physical activities was associated with the risk of developing AD or the rate of cognitive decline. This suggests that the association between cognitive activity and the risk of disease reflects mental stimulation rather than a nonspecific result of being active.

Bottino^51^ found that reading newspapers and books, watching TV, and physical exercise significantly decreased the odds ratio for dementia. Other Brazilian community dwelling studies^50,77-79^ found that engagement in social activities decreased cognitive impairment rates. On the other hand, Baldivia and Bueno80 found no protective effect of engagement in cognitively stimulating activities on the cognitive function of healthy elders in a cohort study.

The mechanisms underlying engagement in cognitive activity and maintenance of cognitive function in the elderly could be explained by the “use and disuse” adage, affirming that changes in everyday experience and activity patterns may result in disuse and atrophy of cognitive processes and skills.^81^ Another possible explanation is the positive correlation between level of cognitive activity and level of cognitive function.^73^ Consequently, systematic engagement in cognitive activities could increase the efficiency and flexibility of neural systems underlying cognitive functions, making these abilities more efficient and less vulnerable to disruption by AD pathology^57^ or reversing age-related cognitive alterations.^3,72,75,82^

### 5.3. Occupation

In spite of the fact that most people spend a substantial portion of their lives at work, understanding of the relationship between occupational activities and cognition is limited. It has been difficult to establish whether occupational experience is an isolated protective factor against cognitive impairment given that occupation is related to levels of education and literacy.^82^ Moreover, education and occupation are related to patterns of cognitive activity in early life and throughout adulthood.^76^

Occupation can be considered non-formal education because it represents a lifelong type of training and possibly the development of specialized skills. The effects of complex work on cognition could be interpreted as facilitating cognitive performance by motivating individuals to perform demanding cognitive operations on a daily basis^83^, or as an action of neuronal plasticity.^84^ It has therefore been theorized that occupation has a protective role on intellectual functioning, suggesting that this variable could be a measure of CR.^85^

Different methodologies have been used to analyze the link between complexity of work and intellectual functioning. National census classifications have been used to define occupation codes and to categorize them in studies conducted in a variety of cultural settings, including France,^86^ the UK,^5^ Sweden,^87^ Taiwan,^88^ the United States^56,89-92^ and Brazil.^80^

Other studies have used national classifications to describe occupation as a first analysis, followed by division of labor according to complexity level and occupation. For example, Stern et al.^56,90^ divided occupations requiring unskilled/semi-skilled, skilled trade or craft, and clerical functions as a low level of complexity; while business manager, government and professional/technical functions were classified as having a high level of complexity. In addition, stratified ordinal value questionnaires have also been used to evaluate job demands.^5,19,92^

Instruments designed to measure each type of demand required by an occupation provide more accurate information about the complexity and involvement of cognitive functions of a profession. This is the case with instruments that classify the complexity of work in domains such as data, people and things ^83,92^ or multiple domains such as the Job Content Questionnaire developed by Karasek ^93^ and adapted to the Brazilian population by Araujo.^94^

Several studies have shown the influence of occupation on cognitive performance among healthy subjects. Schooler et al.^95^ investigated how substantially complex work affected the intellectual functioning of healthy volunteers in a 30-year longitudinal study, and whether this relationship had a function among older workers. The authors found that the level of complexity of an occupation continued to affect the level of intellectual functioning in the same manner as had occurred when the volunteers were 20 and 30 years younger, suggesting that both these variables had reciprocal effects on one another.

Bosma et al.^96^ found that the complexity of work – measured based on the subjects’ estimation of the level of mental demand, concentration and precision required – and the amount of time spent working under pressure were related to reduced risk of developing cognitive impairment three years later and that this relationship was independent of age.

In a cohort of 3,777 aged French people, logistic regression analysis showed that after controlling for age, sex and educational level, farm workers, domestic service employees and blue-collar workers had a higher risk of cognitive impairment than did individuals with an intellectual occupation.^86^ Occupations such as trade, technical and service occupations^97^ or agriculture, craft, plant and machine operators and assemblers^88^ have also been associated with poor cognitive performance.

Ansiau et al.^91^ analyzed the relationship between work-related cognitive stimulation, aging and health in a cross-sectional study. A multiple linear regression analysis showed a significant positive correlation between work-related cognitive stimulation and cognitive performance on memory, processing speed and attention tests, even after controlling for possible confounding factors (e.g. educational level, gender and score for participation in cognitively stimulating activities outside work). They also found that cognitive stimulation only protected against effects of aging on attention test performance. This study did not establish a causal relationship between cognitive performance and engagement in a stimulating work environment, making it unclear if higher cognitive performance is a consequence of a rich work environment. According to Richards et al.,^98^ influencing factors not only determine cognitive ability at any given age, but also augment ability over time. In this sense, when taking prior ability into account, the influence of work complexity as a measure of CR adds variance to later cognitive function. Some studies have shown that occupation contributes to variance in fluid reasoning5 and to better cognitive performance in later life, independent of related factors such as education and intelligence.^89^ Baldivia and Bueno^80^ also found that work level complexity was associated with better performance on the Rey Complex Figure copy (a measure of executive planning) and that this association was independent of confounding variables (e.g. education level, intelligence, socioeconomic level and engagement in cognitively stimulating activities). The authors concluded that complexity of work as a measure of CR could be associated with the maintenance of executive functions in healthy people, delaying the expression of age-related cognitive decline.

Occupation has also been linked to the incidence and course of AD. A classical study by Stern et al.^57^ indicated that higher lifetime educational level and occupation (e.g. manager, professional, technician) were associated with a reduced risk for incident dementia, in contrast to lower educational level and occupation (e.g. unskilled, semi-skilled skilled trade, clerical/office worker). In 1999, Stern et al.^56^ compared performance on memory tests between AD and control groups yearly for four visits. They divided the groups into high and low levels of education and occupation and found a more rapid decline in memory scores in patients with higher educational and higher occupational attainment only in the group with low initial scores. This result could be interpreted as an expression of cognitive reserve: that is, as patients with higher educational and occupational attainment have more cognitive reserve, greater pathology is required before memory begins to be affected. When CR is depleted however, a rapid increase in the rate of clinical decline is observed.

According to Li et al.,^88^ occupation could be a better long-term predictor of cognitive decline than education. Despite the fact that occupation and education level have synergistic effects,^65^ occupation represents an indicator of social class and socioeconomic inequalities.

Finally, an understanding of the variables related to the reserve concept suggests that it must be considered a multidimensional entity. Brain reserve and cognitive reserve might contribute in different ways – although overlapping to some degree – toward a better understanding of the impact of protective factors on cognitive function. Moreover, the differentiation of reserve and neural compensation could have practical effects for functional imaging.

Research in CR requires longitudinal studies in order to understand how variables affect cognitive performance throughout life. An understanding of the variables that can modify cognitive function throughout life shows us the potential benefits of intellectually demanding occupations and education or of intellectually challenging activities in general. All of these could potentially increase an individual’s CR through some set of systematic exposures or interventions. This stimulation could be important for early diagnosis of dementia, as such an approach would help determine prognosis and progression over time and would be crucial for the assessment of any intervention.

In healthy aging, the concept of CR can also be applied to intervention programs which could result in a non-pharmacological approach to reducing the risk of developing AD^6^ or delaying the onset of age-related cognitive decline. In developing countries, the implementation of intervention programs could be one way in which to minimize the heterogeneity of cognitive stimulation that people receive during their lives.

## References

[1] Stern Y, Stern Yaakov (2007). The concept of cognitive reserve: a catalyst for
research. Cognitive Reserve. Theory and applications.

[2] Katzman R, Terry R, DeTeresa R (1988). Clinical, pathological, and neurochemical changes in dementia: a
subgroup with preserved mental status and numerous neocortical
plaques. Ann Neurol.

[3] Stern Y (2002). What is cognitive reserve? Theory and research application of the
reserve concept. J Int Neuropsychol Soc.

[4] Satz P (1993). Brain reserve capacity on symptom onset after brain injury: a
formulation and review of evidence for threshold theory. Neuropsychology.

[5] Staff RT, Murray AD, Deary IJ, Whalley LJ (2004). What provides cerebral reserve?. Brain.

[6] Mortimer JA, Snowdon DA, Markesbery WR (2003). Head circumference, education and risk of dementia: findings from
the nun study.

[7] Stern Y (2006). Cognitive Reserve and Alzheimer Disease. Alzheimer Dis Assoc Disord.

[8] Stern Y, Habeck C, Moeller J (2005). Brain networks associated with cognitive reserve in healthy young
and old adults. Cereb Cortex.

[9] Scarmeas N, Zarahn E, Anderson KE (2003). Cognitive reserve modulates functional brain responses during
memory tasks: a PET study in healthy young and elderly
subjects. Neuroimage.

[10] Rosen AC, Prull MW, O'Hara R (2002). Variable effects of aging on frontal lobe contributions to
memory. Neuroreport.

[11] Grady CL, McIntosh AR, Beig S, Keightley ML, Burian H, Black SE (2003). Evidence from functional Neuroimaging of a compensatory
prefrontal network in Alzheimer's disease. J Neurosci.

[12] Cabeza R, Anderson ND, Locantore JK, McIntosh AR (2002). Aging gracefully: compensatory brain activity in
high-performancing older adults. Neuroimage.

[13] Scarmeas N, Stern Y (2004). Cognitive reserve: implications for diagnosis and prevention of
Alzheimer's disease. Curr Neurol Neurosci Rep.

[14] Alexander GE, Fuery ML, Grady CL GE (1997). Association of premorbid intellectual function with cerebral
metabolism in Alzheimer's disease: implications for the cognitive reserve
hypothesis. Am J Psychiatry.

[15] Stern Y, Alexander GE, Prohovnik I (1995). Relationship between lifetime occupation and parietal flow:
implications for a reserve against Alzheimer's disease
pathology. Neurology.

[16] Stern Y, Alexander GE, Prohovnik I, Mayeux R (1992). Inverse relationship between education and pariotemporal
perfusion deficit in Alzheimer's disease. Ann Neurol.

[17] Kemppainen NM, Aalto S, Karrasch M (2008). Cognitive reserve hypothesis: Pittsburgh Compound B and
fluorodeoxyglucose positron emission tomography in relation to education in
mild Alzheimer's disease. Ann Neurol.

[18] Maguire EA, Gadian DG, Johnsrude IS (2000). Navigation-related structural change in the hippocampi of taxi
drivers. Proc Natl Acad Sci (USA).

[19] Maguire EA, Woollett K, Spiers HJ (2006). London taxi drivers and bus drivers: a structural MRI and
neuropsychological analysis. Hippocampus.

[20] Sole-Padullés C, Bartrés-Faz D, Junque C (2007). Brain structure and function related to cognitive reserve
variables in normal aging, mild cognitive impairment and Alzheimer's
disease. Neurobiol Aging.

[21] Whalley LJ, Deary IJ, Appleton CL, Starr JM (2004). Cognitive reserve and the neurobiology of cognitive
aging. Ageing Res Rev.

[22] Deary IJ, Whalley IJ, Lemmon H, Crawford JR, Star JM (2000). The stability of individual differences in mental ability from
childhood to old age: follow-up of the 1932 Scottish Mental
survey. Intelligence.

[23] Rabitt P, Lowe C (2006). Patterns of cognitive ageing. Psychol Res.

[24] Kramer AF, Willis SL (2002). Enhancing the cognitive ability of older adults. Curr Direc Psychol Science.

[25] Hultsch D, Hertzog C, Small Bj, Dixon RA (1999). Use it or lose it: engaged lifestyle as a buffer of cognitive
decline in aging?. Psychol Aging.

[26] Salthouse TA (1996). General and specific speed mediation of adult age differences in
memory. J Gerontol Psychol Sci.

[27] West MJ (1996). Regionally specific loss of neurons in the aging human
hippocampus. Neurobiol Aging.

[28] Schretlen D, Pearlson GD, Anthony JA (2000). Elucidating the contributions of processing speed, executive
ability, and frontal lobe volume to normal age-related differences in fluid
intelligence. J Int Neuropsychol Soc.

[29] Buckner RL (2004). Memory and Executive Function in Aging and AD: Multiple factors
that cause decline and reserve factors that compensate. Neuron.

[30] Raz N, Gunning FM, Head D (1997). Selective aging of the human cerebral cortex observed in vivo:
differential vulnerability of the prefrontal gray matter. Cereb Cortex.

[31] Raz N, Craik FI, Salthouse TA (2000). Aging of the brain and its impact on cognitive performance:
integration of structural and functional findings. Handbook of aging and cognition - II.

[32] De Carli C, Murphy DG, Gillette JA (1994). Lack of age-related differences in temporal lobe volume of very
healthy adults. AJNR.

[33] Salat DH, Kaye JA, Janowsky JS (2001). Selective preservation and degeneration within the prefrontal
cortex in aging and Alzheimer disease. Arch Neurol.

[34] Good CD, Johnsrude IS, Ashburner J, Herson RN, Friston KJ, Frackowiak RS (2001). A voxel-based morphometric study in ageing in 465 normal adult
human brains. Neuroimage.

[35] Van Petten C, Plante E, Davidson PS, Kuo TY, Bajuscak L, Grisky EL (2004). Memory and executive function in older adults: relationships with
temporal and prefrontal gray matter volume and white matter
hyperintensities. Neuropsychologia.

[36] Den Heijer T, Geerlings MI, Hoebeek FE, Hofman A, Koudstaal PJ, Breteler MMB (2006). Use of hippocampal and amygdalar volumes on magnetic resonance
imaging to predict dementia in cognitively intact elderly
people. Arch Gen Psychiatry.

[37] Van Der Werf YD, Tisserand DJ, Visser PJ (2001). Thalamic volumes predicts performance on tests of cognitive speed
and decreases in healthy aging. A magnetic resonance imaging- based
volumetric analysis. Brain Res cogn Brain Res.

[38] Mattson MP, Chan SL, Duan W (2002). Modification of brain aging and neurodegenerative disorders by
genes, diet and behavior. Physiology.

[39] Gage FH (2000). Mammalian neural stem cells. Science.

[40] Trachtenberg JT, Chen BE, Knott GW (2002). Long-term in vivo imaging of experience-dependent synaptic
plasticity in adult cortex. Nature.

[41] Kempermann G, Gast D, Gage FH (2002). Neuroplasticity in old age: sustained fivefold induction of
hippocampal neurogenesis by long term environmental
enrichment. Ann Neurol.

[42] Lazarov O, Robinson J, Tang YP (2005). Environmental enrichment reduces Abeta levels and amyloid
deposition in transgenic mice. Cell.

[43] LoGiudice D (2002). Dementia: an update to refresh your memory. Intern Med J.

[44] Nitrini R, Caramelli P, Herrera EJr (2004). Incidence of dementia in a community-dwelling Brazilian
population. Alzheimer Dis Assoc Disord.

[45] Prince M (1997). The need for research on dementia in developing
countries. Trop Med Int Health.

[46] Lopes MA, Bottino CMC (2002). Prevalência de demência em diversas regiões
do mundo. Análise dos estudos epidemiológicos de 1994 a
2000. Arq Neuropsiquiatr.

[47] Mangone CA, Arizaga RL (1999). Dementia in Argentina and other Latin-American countries: an
overview. Neuroepidemiology.

[48] Prince M (2000). The 10/66 Dementia Research Group. Dementia in developing
countries. A Consensus statement from the 10/66 dementia research
group. Int J Geriat Psychiatry.

[49] Herrera JR E, Caramelli P, Nitrini R (1998). Estudo epidemiológico populacional de demência na
cidade de Catanduva - estado de São Paulo- Brasil. Rev Psiq Clin.

[50] Herrera JE, Caramelli P, Silveira ASB, Nitrini R (2002). Epidemiological survey of dementia in a community-dwelling
Brazilian population. Alzheimer Dis Assoc Disord.

[51] Bottino CMC (2007). Prevalência de comprometimento cognitivo e demência em
três distritos do município de São Paulo.

[52] Gilleard CJ (1997). Education and Alzheimer's disease: a review of recent
international epidemiological studies. Aging Ment Health.

[53] Rosa TEC, Benício MHD, Latorre MRDO, Ramos LR (2003). Fatores determinantes da capacidade funcional entre
idosos. Rev Saude Publica.

[54] Geerlings MI, Deeg DJH, Schmand B, Lindboom J, Jonker C (1997). Increased risk of mortality in Alzheimer's disease patients with
higher education? A replication study. Neurology.

[55] Geerlings MI, Deeg DJH, Pennix BWJH (1999). Cognitive reserve and mortality in dementia: the role of
cognition, functional ability and depression. Psychol Med.

[56] Stern Y, Albert S, Tang M-X, Tsai W-Y (1999). Rate of memory decline in AD is related to education and
occupation: Cognitive Reserve?. Neurology.

[57] Wilson RS, Bennett DA, Gilley DW, Becket LA, Bames LL, Evans DA (2000). Premorbid reading activity and patterns of cognitive decline in
Alzheimer Disease. Arch Neurol.

[58] Scarmeas N, Albert SM, Manly JJ, Stern Y (2006). Education and rates of cognitive decline in incident Alzheimer's
disease. J Neurol Neurosurg Psychiatry.

[59] Lee JH (2007). Understanding cognitive reserve through genetics and genetic
epidemiology. Yaakov Stern. Cognitive Reserve. Theory and
applications.

[60] Lyketsos CG, Chen LS, Anthony JC (1999). Cognitive decline in adulthood: An 11.5-year follow-up of the
Baltimore Epidemiologic Catchment Area study. Am J Psychiatry.

[61] Starr JM, Deary IJ, Inch S (1997). Blood pressure and cognitive decline in healthy old
people. J Human Hypertens.

[62] Arbuckle TY, Maag U, Pushkar D, Chaikelson JS (1998). Individual differences in trajectory of intellectual development
over 45 years of adulthood. Psychol Aging.

[63] Christensen H, Korten AE, Jorm AF (1997). Education and decline in cognitive performance: Compensatory but
not protective. Int J Geriatr Psychiatry.

[64] Hototian SR, Lopes MA, Azevedo D (2008). Prevalence of cognitive and functional impairment in a community
sample from São Paulo, Brazil. Dement Geriatr Cogn Disord.

[65] Le Carret N, Lafont S, Mayo W, Fabrigoule C (2003). The effect of education on cognitive performances and its
implication for the constitution of the cognitive reserve. Dev Neuropsychol.

[66] Ostrosky-Solis F, Ardila A, Rosselli M, Lopez-Arango G, Uriel-Mendoza V (1998). Neuropsychological Test Performance in Illiterate
Subjects. Arch Clin Neuropsychol.

[67] Alexander G, Furey ML, Grady CL (1997). Association of premorbid intellectual function with cerebral
metabolism in Alzheimer's disease: Implications for the cognitive reserve
hypothesis. Am J Psychiatry.

[68] Manly JJ, Schupf N, Tang MX (2007). Literacy and cognitive decline among ethnically diverse
elders. Cognitive Reserve: Theory and Applications.

[69] Manly JJ, Touradji P, Tang M-X, Stern Y (2003). Literacy and memory decline among ethnically diverse
elders. J Clin Exp Neuropsychol.

[70] Bennett DA, Wilson RS, Schneider JA, Evans DA, Mendes de Leon CF, Arnold SE (2003). Education modifies the relation of AD pathology to level of
cognitive function in older persons. Neurology.

[71] Evans DA, Hebert LE, Beckett LA (1997). Education and other measures of socioeconomic status and risk of
incident Alzheimer's disease in a defined population of older
persons. Arch Neurology.

[72] Katzman R (1993). Education and prevalence of dementia and Alzheimer's
disease. Neurology.

[73] Wilson RS, Bennett DA, Beckett LA (1999). Cognitive activity in older persons from a geographically defined
population. J Gerontol B Psychol Sci Soc Sci.

[74] Wilson RS, Barnes LL, Krueger KR, Hoganson G, Bienias JL, Bennett DA (2004). Early and late life cognitive activity and cognitive systems in
old age. J Int Neuropsychol Soc.

[75] Friedland RP, Fristch T, Smyth K (2001). Patients with Alzheimer's disease have reduced activities in
midlife compared with healthy control-group members. PNAS.

[76] Wilson RS, Mendes de Leon CF, Barnes LL (2002). Participation in cognitively stimulating activities and risk of
incident Alzheimer disease. J Am Med Assoc.

[77] Hototian SR, Lopes MA, Bustamante SEZ (2005). Identification of dementia subjects in three districts of
São Paulo city, Brazil. Int Psychogeriatr.

[78] Cerqueira ATAR, Lebrão ML, Duarte YAO (2003). Deterioração cognitiva e
depressão. O Projeto SABE no Município de São Paulo: uma abordagem
inicial.

[79] Laks J, Batista EM, Guilherme ER (2005). Prevalence of cognitive and functional impairment in
community-dwelling elderly: importance of evaluating activities of daily
living. Arq Neuropsiquiatr.

[80] Baldivia B, Bueno OFA (2008). Influência da ocupação profissional na
formação de Reserva Cognitiva de idosos
saudáveis.

[81] Salthouse TA, Salthouse TA (1991). Disuse interpretations. Theoretical perspectives on cognitive aging.

[82] Helmer C, Letenneur L, Rouch I (2001). Occupation during life and risk of dementia in French elderly
community residents. J Neurol Neurosurg Psychiatry.

[83] Schooler C, Mulatu MS, Oaetes G (2004). Occupational self-direction, intellectual functioning, and self-
directed orientation in older workers: findings and implications for
individuals and societies. Am J Sociol.

[84] Stern N, Scarmeas N (2004). Cognitive reserve: implications for diagnosis and prevention of
Alzheimer's disease. Curr Neurol Neurosci Rep.

[85] Scarmeas N (2007). Lifestyle patterns and cognitive reserve. Yaakov Stern. Cognitive
Reserve. Theory and applications.

[86] Dartigues JF, Gagnon M, Letenneur L (1992). Principal lifetime occupation and cognitive impairment in a
French elderly cohort (Paquid). Am J Epidemiol.

[87] Andel R, Karholt I, Parker MG, Thorslund M, Gatz M (2007). Complexity of primary lifetime occupation and cognition in
advanced old age. J Aging Health.

[88] Li Chung- Yi, Wu SC, Sung F-C (2002). Lifetime principal occupation and risk of cognitive impairment
among the elderly. Industrial Health.

[89] Potter GG, Helms MJ, Plassman BL (2008). Associations of job demands and intelligence with cognitive
performance among men in late fife. Neurology.

[90] Stern Y, Gurland B, Tatemichi TK, Tang MX, Wilder D, Mayeux R (1994). Influence of education and occupation on the incidence of
Alzheimer's disease. J Am Med Assoc.

[91] Ansiau D, Marquié JC, Soubelet A, Ramos S (2005). Relationships between cognitive characteristics of the job, age,
and cognitive efficiency. Int Congress Serie.

[92] Andel R, Vigen C, Mack WJ (2006). The effect of education and occupational complexity on rate of
cognitive decline in Alzheimer's patients. J Int Neuropsychol Soc.

[93] Karasek RA (1979). Job demands, job decision latitude, and mental strain:
implications for job design. Adm Sci Q.

[94] Graça CC, Araújo E (2003). Estresse ocupacional e saúde: contribuições
do Modelo Demanda-Controle. Cienc Saude Coletiva.

[95] Schooler C, Mulatu MS, Oates G (1999). Continuing effects of substantively complex work on the
intellectual functioning of older workers. Psychol Aging.

[96] Bosma H, Van Voxtel MPJ, Pounds R, Houx P, Burdorf A, Jolles J (2003). Mental work demands protect against cognitive impairment: MAAS
prospective cohort study. Exp Aging Res.

[97] Jorm AF, Rodgers B, Henderson AS (1998). Occupation type as a predictor of cognitive decline and dementia
in old age. Age Aging.

[98] Richards M, Deary J (2005). A life course approach to cognitive reserve: a model for
cognitive aging and development?. Ann Neurol.

